# A Survey of Factors Associated with the Utilization of Community Health Centers for Managing Hypertensive Patients in Chengdu, China

**DOI:** 10.1371/journal.pone.0021718

**Published:** 2011-07-07

**Authors:** Yun Chai, Hancheng Xu, Wenxin Wang, Bing Liu, Dehua Yang, Hong Fan, Fujian Song, Zuxun Lu

**Affiliations:** 1 School of Public Health, Tongji Medical College, Huazhong University of Science and Technology, Wuhan, Hubei, People's Republic of China; 2 Faculty of Public Administration, Hubei University of Medicine (original Yunyang Medical College), Shiyan, Hubei, People's Republic of China; 3 Fourth People Hospital of Chengdu, Chengdu, Sichuan, People's Republic of China; 4 Faculty of Health, University of East Anglia, Norwich, East Anglia, United Kingdom; 5 International Exchange and Cooperation Office, Wuhan University of Science and Technology, Wuhan, Hubei, People's Republic of China; University Institute of Social and Preventive Medicine, Switzerland

## Abstract

**Background:**

For decades the development of community health services has been emphasized in China to cope with the growing burden of chronic diseases by providing basic medical services. This survey aims at investigating factors associated with the use of Community Health Centers (CHCs) for the management of hypertensive patients in Chengdu, China.

**Methods:**

We used a systematic sampling method to select 2,030 patients with hypertension or diabetes registered in 29 CHCs in Chengdu in 2007. Researchers interviewed patients who consented to participate at their home. This paper reports findings from the survey of 1,716 hypertensive patients with completed questionnaires. Univariate analyses and multiple logistic regression analyses were conducted to explore factors influencing the use of CHCs for the management of hypertensive patients.

**Results:**

81.4% of hypertensive patients regularly used CHCs for hypertension monitoring and treatment in Chengdu. Univariate analyses indicated that use of CHCs was associated with the education level, occupation, types of medical insurance, Body Mass Index(BMI), patients' knowledge on hypertension, awareness of CHCs functions, satisfaction of the service of CHCs. Multiple regression analyses found that use of CHCs was positively associated with the following factors: the Urban Resident Basic Medical Insurance(URBMI), knowledge on blood pressure, awareness of the sites in CHCs to measure blood pressure, awareness of having to take life-long antihypertensive medicine once the treatment started, awareness of the health records registration in CHCs, regular follow up, improved convenience of seeing doctor. Patients with professional job were less likely to use the services of CHCs.

**Conclusions:**

The use of CHCs for hypertension management could be increased by improving residents' knowledge on the monitoring and treatment of hypertension, and the awareness of CHCs functions. The CHCs could play an important role in providing medical care to low-income, unemployed and other disadvantaged patients with hypertension.

## Introduction

Along with the rapid socio-economic development and demographic changes, the burden of the chronic non-communicable diseases has been increasing in China [1]. The proportion of deaths from chronic non-communicable diseases in all deaths in China increased from 76.5% in 1991 to 82.5% in 2004 [2], and 70% of disability-adjusted life-years lost in China was due to chronic conditions [3]. Therefore, health systems reform in China needs to be orientated to the prevention and management of chronic diseases [4], [5].

The important roles of community health services for the management of patients with chronic diseases have been increasingly recognized [6], [7]. To meet the basic needs of the population for health care and to achieve health equity, the Chinese government in 1997 issued a document “Decisions on Health Service Reform and Development” [1]. The document endorsed the development of community health services for all residents in China. After special documents were enacted in 2002 and 2006, Chinese government has invested heavily in the development of the community health services [8]. The objectives, key players, target populations and essential functions of the community health services have now become clearer [9].

There are currently two types of Community Health Institutions (CHIs) in China: Community Health Centers (CHCs) and Community Health Stations (CHSs). Each CHC occupies a building area of more than 1,000 square-meters, has less than 50 inpatient beds, and provides medical and preventive services to 30,000 to 50,000 residents. The CHS is smaller than a CHC, occupying a building area of more than 150 square-meters, providing outpatient services to around 5,000 residents, and without inpatient beds.

In developed countries CHIs are usually the first point of contact for patients, and there is a two-way patient transferring system. General practitioners (GPs) in CHIs can refer patients to hospitals to be treated by specialists. Simultaneously hospitals can transfer patients who are recovering to CHIs. However, the two-way patient transferring system in China is still in the initial stage. An operational “gatekeeper” system has not been well established in Chinese health service systems. In addition, many residents believe that the quality of services provided by CHCs is low [9], [10]. Therefore, the overuse of high level hospitals and the underuse of primary care services by patients have been widespread in China [11]–[13].

The Urban Health and Poverty Project (UHPP) between 2001 and 2007 in Chengdu was a collaborative project between the Chinese government and the government of the United Kingdom [14]. The project aimed at developing community health services and providing medical aid to the poor, which has led to considerable improvements in the management of chronic diseases in Chengdu [14]. Measures taken to prevent and manage chronic diseases included health education, the establishment of registers of patients with chronic diseases, and information management. Health promotion and prevention activities included the distribution of health education booklets, weekly workshops on selected health topics in CHCs, the increased communication between GPs and patients, media campaigns via television and newspapers to promote healthy life-styles, and prohibition of smoking in public places, and so on. Each community was divided into several sub-districts, and GPs and community nurses were assigned to each sub-district to monitor and manage patients with chronic conditions.

To investigate factors associated with the use of CHCs for the management of patients with hypertension, we conducted a survey of hypertensive patients registered in CHCs in three administrative districts of Chengdu in 2007. In this paper, we report findings from this survey, and provide policy recommendations for the management of hypertensive patients in community health services.

## Methods

The study was approved by the Research Ethics Committee in Tongji Medical College, Huazhong University of Science and Technology, Wuhan, China (Approval form S1). In addition, the researchers obtained consent from all participants involved in our study. We provided detailed information on the study to eligible patients and included only those who consented to participate.

The diagnosis of hypertension was based on the Chinese Guidelines for the Prevention and Treatment of Hypertension [15]. According to the Chinese Guidelines, hypertension is diagnosed when systolic/diastolic blood pressure is higher than 140/90 mmHg. The Chinese Guidelines uses term “high-normal” when blood pressure is 120–139/80–89 mmHg, rather than “pre-hypertension” as in the JNC7 NHLBI Guidelines, in order to avoid unnecessary panic in people with this level of blood pressures.

In this survey, we designed and used a questionnaire (Questionnaire S1) to collect information from patients on demographic and socio-economic characteristics, utilization of CHCs, the awareness and knowledge on the management of hypertension, and the awareness of his/her health records being registered in CHCs. The health record of a patient is a comprehensive document that records her/his life-long heath information including personal details, results of health checkup, prevention and treatment care received, regular monitoring follow-ups, and so forth.

In 2007, the total population of urban area in Chengdu city is 4.85 million. There are 2.31 million residents in the three districts (Jinjiang, Qingyang and Wuhou) investigated. The total number of patients with chronic diseases in urban area of Chengdu city is 679.29 thousands and the total number of the patients registered in CHCs is 397.4 thousands in 2007. We selected three districts from all the six administrative districts in Chengdu city in November 2007. The level of the socio-economic development was taken into consideration in the selection of study districts. One selected district had a relatively high level of socio-economic development, one had medium and another had low level of the development. There are a total of 29 CHCs in these selected districts. A patient can only register and receive services in one CHC. In a database of all registered patients with chronic diseases, the names of patients were listed according to the time of their registration, and each patient has been randomly assigned a registration number. Using the systematic sampling method, we started from the beginning of the lists and selected 70 hypertensive or diabetic patients with an even registration number from each CHC.

The sample size of this study was based on the method recommended by the 3^rd^ National Health Services Survey in China, which suggested that a sample of 600 households and around 2,040 people would have satisfactory representative of the target population in Chengdu city [16]. A total of 2,030 patients were eventually included in this survey, and 2,028 valid questionnaires (including 1,716 hypertension cases) were completed. This paper presents only findings from the survey of 1,716 hypertensive patients.

After a patient consented to participate, a trained investigator interviewed the patient face to face in the evening at the patient's home. The inspectors and the investigators daily checked the collected questionnaires to correct the logic errors of answers and try their best to replenish the blank items during the survey. According to the rechecking ratio (5%) used in the 3^rd^ National Health Services Survey of China [16], the inspectors randomly selected 5% from the finished questionnaires and contacted the patients by telephone to verify the validity of the completed questionnaires at the end of the survey.

Data were processed by using Epi data 3.1 and analyzed by using software SPSS 12.0 (SPSS Incorporated, Chicago, IL, USA). At first, the analyses of demographic and social characteristics of the hypertensive patients were conducted (Table 1), then univariate analyses were conducted to examine the association between the utilization of CHCs for the management of hypertension and influencing factors. Then we used a non-conditional multiple logistic regression model to investigate factors associated with the utilization of CHCs by hypertensive patients. Because of the small sample size, some variables were combined in the analysis. For example, divorce and widower were combined as a marital variable, and management or marketing personnel and manager were combined as an occupational variable. Patients were categorized into three age groups: less than 50, 50∼69, and 70 years of age or older. The regular utilization of CHCs for the management of hypertension was used in the analysis as the response (or dependent) variable, which was defined as that the patients visited a CHC for monitoring or treating hypertension at least once per month. Independent variables included gender, age, educational level, occupation, marital status, medical insurance, BMI, knowledge on hypertension, compliance with the prescription, awareness of health records in CHCs, being regularly followed up and satisfaction of the service of CHCs. The analysis of dummy-variables within each category was based on the arbitrarily decided reference (usually the first level of the variable in the questionnaire, see Table 2 and Table 3 for details). In the logistic regression model, we adopted the Enter Method to achieve a final model. The standard for the variable inclusion was based on SLE = 0.05, and the exclusion standard was SLS = 0.10.

**Table 1:** Demographic and social characteristics of hypertensive patients.

Variable	Categories	Cases	%
**Total**		1716	100.0%
**Age**	<50 years old	73	4.3%
	Age 50∼69	849	49.5%
	≥70 years old	794	46.3%
**Sex**	Male	690	40.2%
	Female	1026	59.8%
**Education**	Illiteracy and semi-illiteracy	215	12.5%
	Elementary school	468	27.3%
	Junior high school	459	26.7%
	Senior high or vocational school	329	19.2%
	College diploma	117	6.8%
	Bachelor or above	128	7.5%
**Occupation**	Civil servants or teachers	243	14.2%
	Professional technician	248	14.5%
	Management or marketing person	93	5.4%
	Managers	8	0.5%
	Employee/worker/attendant	696	40.6%
	Self-employed	40	2.3%
	The unemployed	283	16.5%
	Others	105	6.1%
**Marital status**	Unmarried	9	0.5%
	Married	1445	84.2%
	Divorce	17	1.0%
	Widower	245	14.3%
**Medical insurance**	UEBMI	829	48.3%
	Government insurance	185	10.8%
	URBMI	236	13.8%
	Self payment	252	14.7%
	NRCMS	139	8.1%
	Medical aid	30	1.7%
	Others	45	2.6%

Notes:

UEBMI – the Urban Employee Basic Medical Insurance.

URBMI– the Urban Resident Basic Medical Insurance.

NRCMS– the New Rural Cooperative Medical Scheme.

**Table 2:** Factors associated with the regular use of CHCs for the management of hypertension (This table shows the statistically significant results of univariate analyses and multiple logistic regression analyses).

Variables	Cases	Use of CHCs (%)	*P* value (univariate) χ2 test	*OR* (95% *CI*)*
**Total**	**1716**	**1397 (81.4%)**		
**Occupation**				
Civil servants or teachers	243	205 (84.4%)	0.045	1
Professional technician	248	200 (80.6%)		0.50(0.25–0.98)**
Management or marketing	101	84 (83.2%)		0.98(0.37–2.61)
Employee/worker/attendant	696	583 (83.8%)		0.73(0.37–1.45)
Self-employed	40	33 (82.5%)		0.84(0.20–3.51)
The unemployed	283	213 (75.3%)		0.47(0.22–1.01)
Others	105	79 (75.2%)		0.76(0.28–2.10)
**Medical insurance scheme**				
UEBMI	829	667 (80.5%)	0.000	1
Government insurance	185	159 (85.9%)		1.05(0.57–1.94)
URBMI	236	195 (82.6%)		1.19(0.70–2.04)**
Self payment	252	221 (87.7%)		1.61(0.93–2.77)
NRCMS	139	97 (69.8%)		0.81(0.40–1.64)
Medical Aid	30	28 (93.3%)		2.96(0.60–14.62)
Others	45	30 (66.7%)		0.44(0.19–1.00)**
**Awareness of the normal blood pressure**				
No	353	228 (64.6%)	0.000	1
Yes	1363	1169 (85.8%)		1.74(1.18–2.55)**
**Awareness of sites for measuring blood pressure free of charge**				
No	159	71 (44.6%)	0.000	1
Yes	1557	1326 (85.2%)		4.96(2.99–8.24)**
**Awareness of having to take life-long antihypertensive medicine**				
No	225	125 (55.6%)	0.000	1
Yes	1491	1272 (88.3%)		3.37(2.21–5.16)**
**Knowing registered health records in CHCs**				
No	131	62 (47.3%)	0.000	1
Yes	1545	1307 (84.6%)		2.92 (1.18–7.20) **
**Being regularly followed up**				
No	128	69 (53.9%)	0.000	1
Yes	1543	1296 (84.0%)		18.16(2.37–138.89)**
**the convenience level of visiting doctors**				
Complicated or very dissatisfied or neutral	148	106(71.62%)	0.000	1
Convenient	1061	839(79.08%)		1.36 (0.74–2.49)
Very Convenient	486	440(90.54%)		1.65(1.22–1.99)**

*: OR>1 indicating a higher percentage of CHC use and OR<1 indicating a lower percentage of CHC use.

**: P<0.05.

**Table 3:** Factors associated with the regular use of CHCs for the management of hypertension (This table shows the statistically significant results of univariate analyses and the statistically non-significant results of multiple logistic regression analyses).

Variables	Cases	Use of CHCs (%)	*P* value (univariate) χ2 test	*OR* (95% *CI*)*
**Total**	**1716**	**1397 (81.4%)**		
**Education level**				
Illiteracy-elementary school	683	540 (79.1%)	0.050	1
Junior high school	459	386 (84.1%)		1.18(0.76–1.83)
Senior high/vocational school	329	264 (80.2%)		0.86(0.51–1.45)
College and above	245	207 (84.5%)		1.15(0.57–2.32)
**BMI** †				
BMI<25	407	310(76.2%)	0.002	1
BMI> = 25	1309	1087(83.0%)		1.44(1.00–2.06)
**Complying with the GP's prescription**				
No	91	45 (49.4%)	0.000	1
Yes	1625	1352 (83.2%)		1.91(0.86–4.24)
**Complying with the regular antihypertensive treatments**				
No	157	92 (58.6%)	0.000	1
Yes	1559	1305 (83.7%)		1.24(0.66–2.33)
**Satisfy the environment of Community Health Center**				
Dissatisfied or very dissatisfied or neutral	179	115(64.25%)	0.000	1
Satisfied	923	742(80.39%)		1.29(0.66–2.52)
Very satisfied	593	528(89.04%)		1.08(0.62–1.87)
**Obtain related health services timely**				
Dissatisfied or very dissatisfied or neutral	259	189(72.97%)	0.000	1
Satisfied	1108	906(81.77%)		0.67(0.33–1.38)
Very satisfied	328	290(88.41%)		1.34(0.84–2.16)
**Doctors teach you the prevention knowledge of hypertension initiatively**				
Dissatisfied or very dissatisfied or neutral	109	63(57.80%)	0.000	1
Satisfied	1025	812(79.22%)		2.11(0.93–4.76)
Very satisfied	561	510(90.90%)		1.49(0.76–2.95)
**Think the service attitude of doctors**				
Dissatisfied or very dissatisfied or neutral	73	43(58.90%)	0.000	1
Satisfied	790	618(78.23%)		1.89(0.87–4.10)
Very satisfied	832	724(87.02%)		1.68(0.82–3.44)
**The doctor's professional skills**				
Dissatisfied or very dissatisfied or neutral	295	207(70.17%)	0.000	1
Satisfied	1089	889(81.63%)		1.30(0.56–3.07)
Very satisfied	311	290(93.25%)		1.00(0.61–1.64)
**Satisfy the medical expenditure level**				
Dissatisfied or very dissatisfied or neutral	314	233(74.20%)	0.000	1
Satisfied	1049	839(79.98%)		2.04(0.82–5.06)
Very satisfied	332	313(94.28%)		0.91(0.54–1.55)
**Satisfy the Community Health Center overall**				
Dissatisfied or very dissatisfied or neutral	184	126(68.48%)	0.000	1
Satisfied	1174	945(80.49%)		1.16(0.45–3.00)
Very satisfied	337	314(93.18%)		1.23(0.65–2.31)

*: OR>1 indicating a higher percentage of CHC use and OR<1 indicating a lower percentage of CHC use.

**: P<0.05.

† : For Adults (over 18 years old) 1. WHO criteria: BMI≥25 overweight; BMI≥30 obese 2. Working Group on Obesity for Chinese (WGOC) criteria: BMI≥24 overweight; BMI≥28 obese.

## Results

The demographic and socio-economic characteristics of the hypertensive patients are shown in Table 1. The average age of the patients was 67.5 years old (SD 10.1, ranging from 29 to 97). The proportion of illiterate or semi-illiterate patients was 12.5%, and the proportion of patients with college or higher education was 14.3%. With respect to occupations, 40.6% of the patients were company employees or factory workers or attendants, 14.2% were government officials or teachers, 14.5% were professional technicians, and 16.5% were unemployed (including the people who had no pension). The main health insurance scheme which covered the most urban residents was the Urban Employee Basic Medical Insurance (UEBMI) (48.3%).

Table 2 and Table 3 shows findings on patients' knowledge on hypertension, compliance with GP's prescription, awareness of health records in CHCs, and regular patient follow-up. Although 79.4% of the 1,716 patients said that they knew the approximate range of normal blood pressure, only 48.9% of them (839/1,716) could point out the correct value. The percentage of knowing the sites in CHCs that provide a free service to measure blood pressure was 90.7%. A total of 1,491 (86.9%) patients knew that antihypertensive medication should be taken for the rest of their lives once the treatments started, and 90.9% of the patients complied with the regular antihypertensive treatments. In addition, 90.1% of the patients knew the registration of their health records in CHCs. 1,549 (90.3%) patients were regularly followed up by their GPs, which was carried out mainly by a home visiting (27.5%) and in clinics or by telephone contacts (70.5%).

The percentage of patients whose blood pressure disorder was regularly monitored and treated in CHCs is also shown in Table 2 and Table 3. Of the 1,716 patients, 81.4% (95% confidence interval: 79.6% to 83.2%) were regularly monitored and treated in CHCs. Results of univariate analyses suggested that the percentage of using CHCs for hypertension management was not significantly associated with age, gender, marital status, methods of follow-up (Table S1). The management of hypertension was statistically significantly associated with the level of education (p = 0.05), occupations (p = 0.045) and medical insurance schemes (p<0.001), although the absolute difference in the percentage between different categories seemed usually small. However, there were statistically significant and considerable differences in the use of CHCs when the patients were divided into different groups by some variables of knowledge or awareness (Table 2 and Table 3).

The final model of the multiple logistic regression analysis included nine significant variables: being a professional technician, taking up the Urban Resident Basic Medical Insurance (URBMI), belonging to “Others” category of the medical insurance scheme, knowing the value of normal blood pressure, awareness of the sites that provided a free service to measure blood pressure, awareness of having to take life-long antihypertensive medication, knowing health records being registered in the CHCs, being regularly followed up, and improved convenience of seeing doctors. Among these eight significant variables, only the occupation of professional technician was negatively associated with the use of CHCs services, and the remaining seven variables were associated with a greater use of CHCs services for hypertension management (Table 2 and Table 3).

## Discussion

It has been estimated that 60% of deaths from cardiovascular diseases in China were attributable to high blood pressure [17]. Data from a nationally representative study in 2004 indicated that only 31% of Chinese patients with hypertension were aware of the condition of their disease, 23.1% were treated with antihypertensive medications, and only 8.0% achieved satisfactory control of hypertension [18]. Because of the UHPP project and the recent development of community health services, the management of patients with hypertension in Chengdu city has been much improved [14]. This survey found that 90.9% of the patients registered in CHCs in Chengdu city in 2007 were regularly treated with antihypertensive medications, which was much higher than the reported national average of 23.1% in 2004.

In China, the development of community health services is still in the early stage. The residents are often not familiar with the services provided in CHCs. For example, the reported percentage of residents who did not know the services provided in CHCs was 38.4% in Zhuhai [11], 25.1% in Jiangsu [19], and 35.0% in Shenzhen [20]. To improve the utilization of CHCs, it is necessary to increase the residents' awareness of the services provided in CHCs. Meanwhile, the actual use of CHCs may also improve the residents' comprehension of CHCs functions. This bi-directional process has been appreciated in China. For example, the offering of the free of charge service to measure blood pressure in Chengdu city aims at attracting hypertension patients to CHCs and to encourage the use of CHCs by patients with chronic diseases. The result from this study confirmed that patients who knew the sites for measuring blood pressure free of charge in CHCs were more likely to use CHCs for the management of hypertension. CHCs are often not the first point of contact by patients for the treatment of hypertension. Publicity campaign to advertise CHCs for the management of chronic conditions have been conducted in Chengdu city. However, some chronic patients in this survey still didn't acquaint the functions of CHCs well. Clearly, more efforts are required to increase the residents' understanding and trust in CHCs. The more the patients understand the functions of CHCs, the more they are likely to use CHCs.

The use of CHCs for the management of hypertension was also high in patients covered by the Medical Aid (93.3%) and patients with self payment (87.7%). Hypertensive patients covered by the URBMI and others had a lower percentage (82.6%, 66.7%) of using CHCs for the management of hypertension. The UEBMI covers employees of and pensioners retired from state-owned companies, civil organizations, and government departments. The participants of the URBMI include those who are not covered by the UEBMI, including students, children, and the unemployed. Others are those who are not covered by the insurances. The URBMI covers the costs of the most inpatient care in CHCs or hospitals, but does not cover the costs of the outpatient care. The costs in CHCs are usually much lower than in hospitals. The relatively low expenses in CHCs have attracted patients with the URBMI. It is possible that more patients may use CHCs for the management of chronic conditions if the costs of outpatient treatments in CHCs could be covered by all medical insurance schemes.

We are unable to explain the finding that patients employed as a professional technician was less likely to use CHCs. A previous study found that CHCs remain the first choice to see a doctor by low-income families and the unemployed [21]. However we observed the relatively low percentage of the use of CHCs by patients belonging to occupational category “Others” or “the unemployed” (75.2% and 75.3% respectively) as well as by patients belonging to “Others” category of the medical insurance scheme (66.7%). The category “Others” for occupation or the medical insurance scheme included mainly patients who were migrants from rural areas. In addition, the percentage of the use of CHCs by low-income patients for the management of hypertension was low. Low-income patients were also less likely to be covered by social insurance systems. To meet the basic health needs of low-income families, the unemployed and other disadvantaged patients, the government should continue to directly invest in the development and running of CHCs, to avoid these health institutions seeking profits from providing basic services. However, the financial investment offered to the CHCs by the government is generally not enough so that the CHCs currently have to depend on the income of the medical services and medicine to compensate their deficit [22].

We found that the use of CHCs for monitoring and treating hypertension by patients was associated with their knowledge on hypertensive disease, the awareness of the register of health records in CHCs, and being regularly followed up. The awareness of the normal blood pressure was specifically used as one indicator of the effect of the health education campaign by government and CHCs. In Chengdu city, the percentage of hypertensive patients knowing the correct value of the normal blood pressure was still low (48.9%). Therefore, more effective health education interventions should be developed. In addition, the development of databases of patients with chronic conditions is fundamental in order to systematically and continuously monitor and treat patients with hypertension. In Chengdu, an effective method for managing patients with hypertension is the regular follow-up of patients by primary care professionals in CHCs. The assignment of GPs to the allocated neighborhood has facilitated the continuity of patient care, regular monitoring, and the provision of advice to residents on the disease control and health promotion.

To cost-effectively control hypertension and other chronic conditions, the further development and increased use of CHCs in China is essential. GPs in CHCs should prescribe effective, safe, low-cost and readily available antihypertensive treatments, and provide advice on healthy life-styles to patients with hypertension. In addition, patient self-management of chronic diseases is important, and needs to be greatly developed in future [6], [23]. An appropriate combination of management by GPs in CHCs and the patient self-management should be one of the priorities to cope with the increasing burden of chronic conditions in China.

## Supporting Information

Approval Form S1 Research Ethics committee approval form.
(PDF)

Questionnaire S1 Questionnaire of utilization situation of Community Health Center for diabetes and hypertension patients.
(DOC)

Table S1 Statistically non-significant results of univariate analyses of factors associated with the regular use of CHCs for the management of hypertension.
(DOC)
