# Supplementary material for: A Survey of Factors Associated with the Utilization of Community Health Centers for Managing Hypertensive Patients in Chengdu, China
Source: PLoS One. 2011 Jul 7;6(7):e21718. doi: 10.1371/journal.pone.0021718 (PMC3131288; doi:10.1371/journal.pone.0021718)
Supplement: Approval Form S1 — Research Ethics committee approval form. (PDF) [file pone.0021718.s001.pdf]

HUAZHONG UNIVERSITY OF
SCIENCE AND TECHNOLOGY

華中科技大學

Wuhan 430074, P. R. China
Tel: (86) 27- 83630652
Fax: (86) 27-83630652

中华人民共和国 湖北武汉

RESEARCH ETHICS COMMITTEE APPROVAL FORM

The Ethics Committee of Tongji Medical College, Huazhong University of Science and Technology (IRB No: FWA00007304) has decided to give final APPROVAL for the study Analysis on monitoring disease status of the hypertension patients in Community Health Centers and its influential factors in Chengdu city, which is conducted by Prof. ZuXun Lu at Social Medicine Department of Public Health School, Tongji Medical College, Huazhong University of Science and Technology. It meets the Declaration of Helsinki and don't impact patient's privacy.

华中科技大学同济医学院药品临床研究基地 医学伦理委员会

Fandian Zeng	[signature]	Oct. 8, 2007
Printed Name	Signature	Date

IEC Chairperson/Designee
