# Supplementary material for: A Survey of Factors Associated with the Utilization of Community Health Centers for Managing Hypertensive Patients in Chengdu, China
Source: PLoS One. 2011 Jul 7;6(7):e21718. doi: 10.1371/journal.pone.0021718 (PMC3131288; doi:10.1371/journal.pone.0021718)
Supplement: Questionnaire S1 — Questionnaire of utilization situation of Community Health Center for diabetes and hypertension patients. (DOC) [file pone.0021718.s002.doc]

**Questionnaire of utilization situation of Community Health Center for diabetes and hypertension patients**

**Finished on:** **Investigator (signature):**

**Recheck on:** **investigation supervisor (signature):**

Dear Sir or Madam:

I am an interviewer of Urban Social-Economic Survey Team, Chengdu city. We are implementing a survey of community health services. If it’s convenient, we hope sincerely know some opinions and suggestions of you as well as your family about community health services. Thanks for your help and your valuable time.

According to the ‘Statistical data pertaining to State secrets must be kept confidential．Single item investigation data concerning any individual or his/her family shall not be divulged without the consent of the said person. the Article 15, Chapter III, Statistics Law of the People's Republic of China.’ We will obey to this law strictly and all your data will be kept confidential. Therefore please do not worry about this. We expect your significant advices. Thank you very much!

**I. Basic information**

1. Community name： Community: Community Health Service Centre

2. Patient name： Telephone: (District Code: )

3. Age: years old

4. Sex: （1）Male （2）Female

5. Your Education level is：

(1) Illiterates and Semi-illiterates (2) Elementary school (3)Junior High school (4) Senior High school or vocational High school (5) College Diploma ⑹ Bachelor or above

6. Your occupation is：

(1) Government officer or teacher (2) Professional technician (3) Middle-class Management or Marketing person (4) Managers (5) Employee/worker/attendant (6) Self-employed (7) The unemployed

7. Marital status is：

(1) Unmarried (2) Married (3) Divorce (4) Widower

8. Payment method of medical expenses is:

(1) The Urban Employee Basic Medical Insurance System (2) the Government Insurance Scheme (3) Self payment (4) the Urban Resident Basic Medical Insurance System (5) New Rural Co-operative Medical System (6) the Medical Aid (7) Others

9. How much was your total medical expenses used in treatment of chronic illness in 2006：

(1)100 RMB-200 RMB (2) 200 RMB -500 RMB (3)500 RMB -1000 RMB (4)1000 RMB—2000 RMB (5)2000 RMB -5000 RMB (6) more than 5000 RMB

10. How much was your total medical expenses used in treatment of chronic illness in the first half of 2007：

(1)100 RMB-200 RMB (2)200 RMB -500 RMB (3)500 RMB -1000 RMB (4)1000 RMB—2000 RMB (5)2000 RMB -5000 RMB (6) more than 5000 RMB

11．Your average medical expense used in treatment of chronic illness every time is RMB.

**The followings questions only need be answered by the hypertension patients**

13. How long have you been diagnosed as hypertension patients?　　 Years.

13.1 Do you accept the monitoring and treatment of your hypertension in the Community Health Centre?

(1) Yes (2) No

13.2 How often do you monitor your hypertension?

(1) One week (2) Two weeks (3) One month (4) More than one month

14. Do you know it can be diagnosed as hypertension how many the blood pressure exceed?

(1) No (2) Yes. it is：　　 mmhg

15. What factors will result in the rising of blood pressure? （Multi options）（ ）

(1) Eating salt more than 6g every day (2) Mental tension (3) Lack of Physical Exercises (4) Inheredity (5) High cholesterol (6) Obesity (7) Over smoking (8) Male>55 years old, Female>65 (9) Diabetes (10) Malnutrition (11) Acalcerosis (12) Over Drinking

16. Does it have the free charge site of measuring blood pressure in your community?

(1) Yes (2) No （3）I don’t know

17. Do you hope the GP to provide door-to-door services to your family? (1) Yes (2) No

18. Do you k now the name of your family’s GP in your community?

(1) Yes（the name is： ）(2) No

19. Do you think you will have to take medicine all your life once accepts medicine treatment?

(1) Yes (2) No (3) I don’t know

20. Whether you take medicine complying with the GP’s advice? (1) Yes (2) No

21. Whether you take medicine regularly complying with the GP’s advice?　 　 (1) Yes (2) No

22. Do you k now the name of the medicine you ever take?

(1) Yes (fill in at least one medicine name: ) (2) No

23. After you are monitored and treated in the Community Health Service Center

(Please answer the following questions)

23.1 Do your daily [dietary](http://www.iciba.com/dietary/) salt [intake](http://www.iciba.com/intake/) decrease?

(1) Yes（Daily intake of salt decrease from gram to gram） (2) No

23.2 Do your daily ethanol intake decrease?

(1) Yes（Daily intake decrease from gram to gram） (2) No

23.3 Do you decrease your daily [smoking](http://www.iciba.com/smoking/) [amount](http://www.iciba.com/amount/)?

(1) Yes（Daily amount from cigarette to cigarette） (2) No

23.4 Have you lost any [weight](http://www.iciba.com/weight/)?

(1) Yes（Decrease from kg to kg compared to last year） (2) No

23.3 Do you do more physical exercises than before? (1) Yes (2) No

23.4 Do you angry less than before? (1) Yes (2) No

23.5 Do you live more regularly than before? (1) Yes (2) No

24. Did your hypertension have been controlled？(Which means the blood pressure is lower than 140 mmhg /90 mmhg lasting for more than half a year):

(1) Yes (2) No (the main reason is 　 　　　 )（3）I don’t know

**The followings only need be answered by diabetic patients**

13. How long have you been diagnosed as diabetic patients?　 　 Years.

13.1 Do you accept the monitoring and treatment of your diabetes in the community health centre?

(1) Often (2) Some times (3) Occasional (4) Never

13.2 How often do you monitor your diabetes?

(1) One week (2) Two weeks (3) One month (4) More than one month

14. Do you know it will be diabetic how many **the level of the fasting serum glucose exceed through measuring finger blood**?

(1) No 　 (2) Yes specify: mmol/L

15. Do you know what the symptoms of diabetes are? (Multi options)

(1) Drink water too much (2) Polyuria (3) Eat too much (4) Loss of weight 　（5）I don’t know

16. Do you hope the GP to provide door-to-door services to your family? (1) Yes (2) No

17. Do you know the name of your family’s GP in your community?

(1) Yes（the name is： ） (2) No

18. Do you think you will have to take medicine all your life once accept medicine treatment

(1) Yes (2) No (3) I don’t know

19. Do you take medicine complying with the GP’s advice? (1) Yes (2) No

20. Do you take medicine regularly complying with the GP’s advice?　 　 (1) Yes (2) No

21. Do you know the name of the medicine you ever take?

(1) Yes (specify one at least: ) (2) No

22. After you are monitored and treated in the Community Health Service Center

(Please answer the following questions)

22.1 Do you have controlled your food-intake?

(1) Strictly (2) Occasionally (3) No

22.2 Do you do exercises more than before?　 　 (1) Yes (2) No

23. Have your symptoms of diabetes been controlled? (the standards are that **the level of fasting serum glucose is** lower than 7 mmol/L lasting for more than half a year or the level of **serum glucose after eating is** lower than 11 mmol/L lasting for more than half a year):

(1) Yes (2) No（the main reason is 　　　　 ）（3）I don’t know

**II. The management condition in your Community Health Centers（both hypertension patients and diabetic patients answer）**

1. How do the Community Health Center find your illness of hypertension or diabetes) ?

(1) Follow-up (2) Health education (3) Physical examination （4）Door-to-door investigation

(5)Visiting to the doctors initiatively

2. Do the Community Health Center file your health records?　 　(1) Yes (2) No

3. Do the GP contact you regularly? 　 　 (1) Yes (2) No

3.1 If yes，the main contact method is：

(1) Door-to-door follow-up (2) Follow-up in clinic or through phone

(3) Follow-up in meeting with all residents

3.2 How often do the GP’s follow-up?　 ,and the average time of follow-up is minutes.

5. How many times have you accept the health knowledge publicity since 2007? It is times

6. How many times have you obtain the free-of-charge medical advices from the Community Health Service Center since 2007? It is times

**III. The satisfaction survey of both hypertension patients and diabetic patients**

1. Do you satisfy the environment of Community Health Center?

(1)Very satisfied (2) Satisfied (3) Neutral (4) Dissatisfied or very dissatisfied

2. How do you think of the convenience level of visiting doctors in the Community Health Center?

(1)Very Convenient （2）Convenient （3）Neutral （4）Complicated （5）Very complicated

3. Can you obtain related timely health services when you have health demand or contact the Community Health Center emergently?

(1) Very Timely （2）Timely （3）Neutral （4）Late （5）Never response

4. Do doctors teach you the prevention knowledge of hypertension or diabetes when you visit the Community Health Center?

（1）Very Actively （2）Actively （3）Neutral （4）Passive （4）Very passive

5. How do you think the service attitude of doctors?

(1) Excellent (2) Good (3) Neutral (4) Bad (5) Very bad

6. How do you evaluate the doctor’s professional skills?

(1) Excellent (2) Good (3) Neutral (4) Bad (5) Very bad

7. Do you satisfy the medical expenditure level in the Community Health Center?

(1)Very satisfied (2) Satisfied (3) Neutral (4) Dissatisfied (5) Very dissatisfied

8. Do you satisfy the Community Health Center overall?

(1)Very satisfied (2) Satisfied (3) Neutral (4) Dissatisfied (5) Very dissatisfied

9. What are your expectations to the Community Health Center?
