# Supplementary material for: A Survey of Factors Associated with the Utilization of Community Health Centers for Managing Hypertensive Patients in Chengdu, China
Source: PLoS One. 2011 Jul 7;6(7):e21718. doi: 10.1371/journal.pone.0021718 (PMC3131288; doi:10.1371/journal.pone.0021718)
Supplement: Table S1 — Statistically non-significant results of univariate analyses of factors associated with the regular use of CHCs for the management of hypertension. (DOC) [file pone.0021718.s003.doc]

Table 3. Statistically non-significant results of univariate analyses of factors associated with the regular use of CHCs for the management of hypertension

| **Variables** | **Cases** | **Use of CHCs (%)** | ***P* value (univariate)**  **χ2 test)** | ***OR* (95% *CI*)*** |
| --- | --- | --- | --- | --- |
| **Total** | **1716** | **1397 (81.4%)** |  |  |
| Age | | | | |
| <50 years old | 73 | 60 (82.2%) | 0.25 | 1 |
| Age 50～69 | 849 | 706 (83.1%) |  | 1.46(0.69-3.09) |
| ≥70 years old | 794 | 631 (79.5%) |  | 1.22(0.57-2.65) |
| Sex | | | | |
| Male | 690 | 571 (82.7%) | 0.24 | 1 |
| Female | 1026 | 826 (80.5%) |  | 1.16(0.84-1.62) |
| Marital status | | | | |
| Unmarried | 9 | 7 (77.8%) | 0.94 | 1 |
| Married | 1445 | 1176 (81.4%) |  | 1.26(0.21-7.65) |
| Divorce or widower | 262 | 214 (81.7%) |  | 1.70(0.27-10.85) |
| Ways of the regular following up | | | | |
| Home follow-up | 427 | 359 (84.1%） | 0.19 | 1 |
| In clinics or telephone | 1095 | 917 (83.7%） |  | 1.40(0.96-2.03) |
| Group workshops | 32 | 23 (71.9%） |  | 1.97(0.71-5.48) |
